# Melatonin as an Efficient and Eco-Friendly Tool to Increase Yield and to Maintain Quality Attributes during Lemon Storage

**DOI:** 10.3390/ijms251810025

**Published:** 2024-09-18

**Authors:** Fátima Badiche-El Hilali, María E. García-Pastor, Juan Miguel Valverde, Salvador Castillo, Daniel Valero, María Serrano

**Affiliations:** 1 Department of AgroFood Technology, Escuela Politécnica Superior de Orihuela (EPSO), Instituto de Investigación e Innovación Agroalimentario y Agroambiental (CIAGRO), University Miguel Hernández, Ctra. Beniel km. 3.2, Orihuela, 03312 Alicante, Spain; fbadiche@umh.es (F.B.-E.H.); jm.valverde@umh.es (J.M.V.); scastillo@umh.es (S.C.); 2 Department of Applied Biology, Escuela Politécnica Superior de Orihuela (EPSO), Instituto de Investigación e Innovación Agroalimentario y Agroambiental (CIAGRO), University Miguel Hernández, Ctra. Beniel km. 3.2, Orihuela, 03312 Alicante, Spain; m.garciap@umh.es

**Keywords:** *Citrus*, yield, quality, phenolic compounds, antioxidant activity, preharvest, postharvest

## Abstract

Lemon fruit (*Citrus limon* (L.) Burm.) is highly appreciated by consumers due to its antioxidant properties and health benefits. However, its shelf life can be limited by various factors, reducing the economy, and thereafter, new strategies to maintain the quality of lemons are necessary. Melatonin is a derivative of tryptamine, which is ubiquitously found in plants and has a wide range of functions regulating numerous physiological processes in plants. During two consecutive harvests, we evaluated the effect of preharvest treatments with melatonin on crop yield and on quality and functional properties of fruit of lemon cv. Verna at harvest and weekly after storage up to 28 days at 2 and 10 °C plus 2 days at 20 °C. Melatonin was applied as foliar spray treatments at dosages of 0.1, 0.3, and 0.5 mM and at three different stages of fruit development. The results showed that melatonin treatment had a positive impact on crop yield as well as in fruit quality parameters, such as firmness, content of bioactive compounds, and antioxidant activity, especially for a 0.5 mM dose. Taking all these effects into account, the application of melatonin along the growth cycle of fruit development could be considered a non-contaminant and eco-friendly tool for improving crop yield and quality of ‘Verna’ lemons at harvest and during postharvest storage.

## 1. Introduction

Originating in Asia, lemon fruit (*Citrus limon* (L.) Burm.) is traditionally consumed for its pleasant taste and perfume and also for its beneficial effects for health, the most prominent ones being antioxidant, anticancer, anti-inflammatory, and antidiabetic, along with neuroprotection and cardiovascular defense [1,2]. These effects are primarily attributed to its rich concentration of bioactive compounds such as phenolic compounds and ascorbic acid [3]. For all these reasons, the lemon has always been valued by consumers, and within the *Citrus*, a genus of the plant family *Rutaceae*, it is the third most produced and consumed species worldwide [4]. Spain is the 7th main lemon fruit producer country around the world, with 863.2 thousand tons in 2022 and 52,600 ha cultivated [5]. This production is mainly located in the eastern regions of Spain (Valencian community and the region of Murcia), in which the lemon crop is a significant source of employment and economic income, with an average trade balance in the last years of ca. 3.2 million euros [6] for the nation. The Verna lemon is among the most widely grown in these regions, mainly due to the great adaptability of this cultivar to the climate of these Spanish areas [7].

While the production and trade of lemons have become increasingly important within the agricultural-food sector, driven by the rising demand from domestic and global markets, substantial difficulties persist concerning the quality and management of this citrus fruit. Challenges include improving production efficiency and sustainability, enhancing the growth and maturation processes, and refining postharvest practices. The progressive loss of quality in fruits and vegetables during transportation and commercialization translates into significant economic losses, posing significant challenges to the industry. The most commonly used method to mitigate the rapid deterioration of these foodstuffs is cold storage [8]. Important quality losses, including a series of depressions in the skin, usually presented in brown color, husk desiccation or decomposition, and firmness loss, occur during post-harvest cold preservation of lemon [9]. Furthermore, titratable acidity, ascorbic acid, and phenolic compounds of the fruit also decrease, leading to a reduction in consumer acceptability in terms of freshness, juiciness, and flavor [10]. Lately, to prevent these undesirable changes and extend shelf life, different preharvest strategies have begun to be developed to improve the quality attributes of lemons at the time of harvesting and maintain them for a longer period during cold storage.

In this scenario, the utilization of several natural elicitor moieties represents a more sustainable alternative to chemicals [11]. Elicitors offer a feasible option to foster a model of modern sustainable agriculture by replacing the reliance on agrochemicals for food. Thus far, there have been no reports suggesting that the use of elicitors, whether of biotic or abiotic origin, causes adverse effects on plants, human health, or the environment [12]. Moreover, fruit crops are highly susceptible to a variety of abiotic stressors, which have been exacerbated by abnormal shifts in climate [13]. Among these, the rise in atmospheric temperature stands as a critical issue, influencing crop yields, food quality, security, availability, and nutrient profiles [14]. In response, it is imperative to implement measures to mitigate climate change effects and pursue effective adaptation strategies. Despite the limitations in the research, the application of natural elicitors, such as γ-aminobutyric acid, has demonstrated potential in mitigating the adverse impacts of climate change on the lemon crop [15].

Melatonin (MEL) was discovered in 1995 in plants belonging to both monocotyledonous and dicotyledonous species and is mainly recognized in animals for its function in controlling sleep [16]. Melatonin plays crucial roles in plants, acting as a growth regulator, antioxidant, and defense mediator. It stimulates seed germination and the growth of roots and shoots, as observed in rice seeds [17]. Additionally, it protects against oxidative stress by enhancing the activity of antioxidant enzymes and reducing reactive oxygen species, aiding plants in tolerating adverse conditions such as salinity [18]. Melatonin additionally regulates the expression of defense-related genes in Arabidopsis plants, preparing them to face pathogens and abiotic stress [19]. Furthermore, it may influence the regulation of circadian rhythms and photoperiods in plants [20]. Overall, MEL is a multifunctional molecule of great interest for biotechnology and agriculture due to its roles in growth, protection, and potential regulation of plants internal biological rhythms.

Moreover, recent studies reported an important role of MEL in controlling the fruit ripening process after preharvest treatment application. Therefore, the use of 0.1 mM melatonin in cherry tomatoes at different growth stages by spraying systems improves postharvest disease resistance [21]. Foliar spray application of melatonin on apricot trees resulted in higher crop yield and increased weight of the fruits [22], as well as increased pigmentation of red pear and sweet cherry by activating the anthocyanin biosynthesis [23,24]. In addition, preharvest 0.1 mM MEL treatment of apricot trees led to fruit with higher-quality attributes during cold or room storage temperatures due to a delay of the ripening process in this climacteric fruit species [25]. Accordingly, postharvest melatonin treatment delayed postharvest ripening in other climacteric fruits, such as cherimoya [26], mango [27], banana [28], and pear [29], throughout the reduction of ethylene production due to decreased ACC-synthase (ACS) and ACC-oxidase (ACO) activities and the expression of their codifying genes. Moreover, pre- and postharvest melatonin treatments have been reported to delay the ripening process in non-climacteric fruit [30,31]. For instance, sweet cherry tree treatment with melatonin, as well as postharvest treatments, let to fruit with delayed ripening during cold storage, which was attributed to enhanced activity of antioxidant systems, both enzymatic and non-enzymatic ones [32,33]. However, the effect of MEL before harvest on the yield and quality characteristics of lemon fruits at harvest or during storage has not been evaluated. Thus, the aim of this research was to evaluate the effect of MEL on lemon quality at harvest and during cold storage. By addressing these issues, results would encourage the development of more sustainable farming methods to increase the value of lemon fruit, benefiting the environment, producers, and consumers.

## 2. Results

### 2.1. Crop Yield

**Figure 1:**
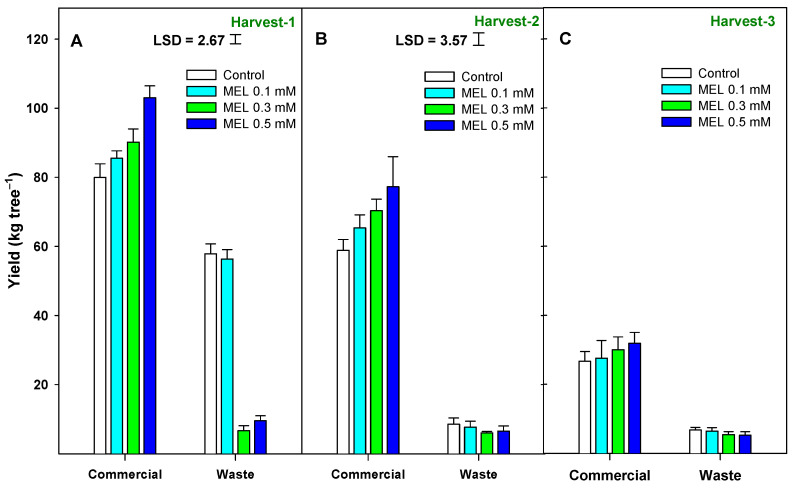
Yield (kg tree^−1^) as affected by preharvest melatonin (MEL) application at three harvesting dates and the two established categories: commercial and waste. Data are the mean ± SE. LSD at *p* < 0.05 for the interaction treatment and fruit categories are shown when such interaction was significant.

**Figure 2:**
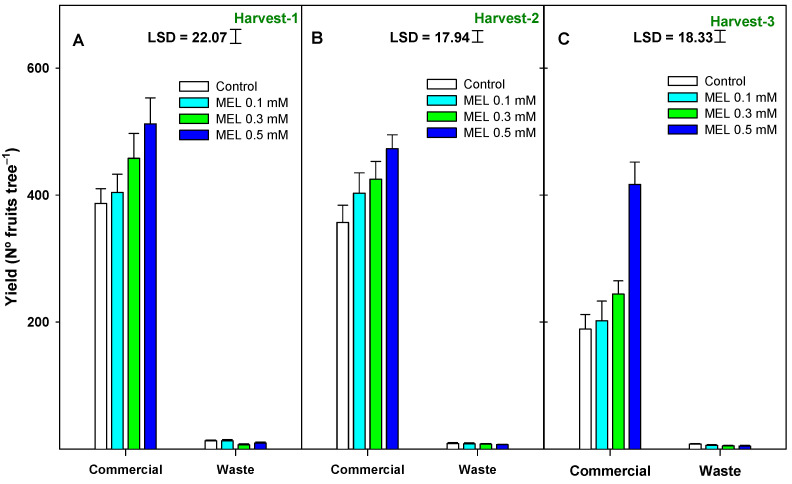
Yield (number of fruits tree^−1^) as affected by preharvest melatonin (MEL) application at three harvesting dates and the two established categories: commercial and waste. Data are the mean ± SE. LSD at *p* < 0.05 for the interaction treatment and fruit categories (commercial or waste) are shown.

Crop productivity and yield were evaluated through three parameters: kilograms of fruit per tree (kg tree^−1^) (Figure 1), the number of lemons per tree (Nº fruit tree^−1^) (Figure 2), and the average fruit weight in grams (Figure S1). ‘Verna’ lemon cultivar is usually harvested between May and July at three different dates. For each picking date, two fruit categories were established: commercial and waste (non-commercial) lemons, according to the marketing procedures. In Figure 1A, it can be observed that in the first harvest, the MEL treatments significantly increased (*p* < 0.001) the yield in kg tree^−1^ of commercial fruit and decreased (MEL 0.3 and 0.5 mM) that of waste (Table S1). In the second harvest (Figure 1B), a similar effect was observed (*p* < 0.05). However, in the third harvest (Figure 1C), there was no significant (*p* ≥ 0.05) difference between the production of the treated trees and the controls (Table S1). In both the first and second harvests, there was a significant interaction (*p* < 0.001 and *p* < 0.01, respectively) between the MEL treatment and the type of fruit harvested on the production in kg (Table S1). 

Next, it was observed that the number of fruits per tree showed the same trend as the crop yield evaluated in kg per tree (Table S1). There was a significant increase (*p* < 0.05) in the number of fruits of MEL-treated trees with respect to control trees (Figure 2A,B). On the other hand, the production type factor was also significant (*p* < 0.001) in the number of fruits per tree, being lower in the proportion of waste fruit in lemon trees treated with MEL 0.3 and 0.5 mM than in controls. In Harvest-3 (Table S1), the same effect as in the two previous harvests for two of the factors studied (treatment and production type) was found but with a higher significance level (*p* < 0.001) in the treatment. In both Figure 1 and Figure 2, the most effective treatment was the MEL 0.5 mM dose. Finally, in Figure S1A–C, which represents the average weight of the fruits, it can be observed that in general, the treatments with MEL had no significant effect (*p* ≥ 0.05) on fruit weight either for commercial or waste fruit, and there wasn’t a significant interaction between both factors.

### 2.2. Fruit Quality Parameters

For each preharvest application (control and MEL at 0.1, 0.3, and 0.5 mM), lemons from Harvest-1 and Harvest-2 were submitted to postharvest storage for one month at two temperature regimes: 2 and 10 °C plus a further period of 2 days at 20 °C (shelf-life, SL). The following quality traits were determined at harvest (day 0) and after 7, 14, 21, and 28 days plus SL: weight loss, respiration rate, fruit firmness, total soluble solids (TSSs), and total acidity (TA). 

**Figure 3:**
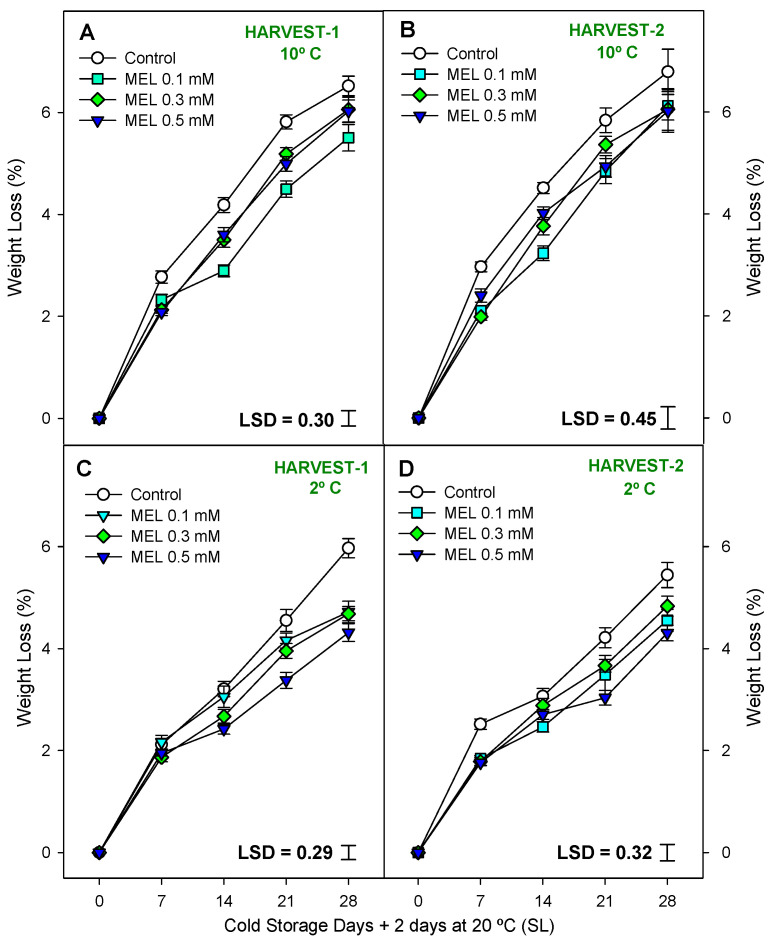
Weight loss (%) during 28 days of storage + shelf-life (SL) at two temperatures (2 and 10 °C) from two harvest dates (HARVEST-1 and HARVEST-2) in lemon fruit as affected by melatonin (MEL) treatment. Data are the mean ± SE. LSD at *p* < 0.05 for the interaction treatment and storage time are shown.

Table S2 shows that weight loss was significantly lower (*p* < 0.001) in the treated fruits (Mel at 0.1, 0.3, and 0.5 mM) compared to the control fruits for both harvests and both temperatures (Figure 3A–D). It was also observed that throughout the storage period, weight losses increased significantly (*p* < 0.001), independently of the treatment and storage temperature, although at 2 °C the losses were lower (Table S2) than at 10 °C. The MEL 0.5 mM treatment was the most effective dose in reducing weight losses in both harvests at 2 °C (4.3 ± 0.2% for Harvest-1 and 2, respectively). Furthermore, the interaction between treatment effect and storage time significantly influenced (Table S2) the weight losses in the four conditions studied, although more so in Harvest-1 at 10 °C and Harvest-2 at 2 °C (*p* < 0.001), according to the LSD values in Figure 3A–D. 

**Figure 4:**
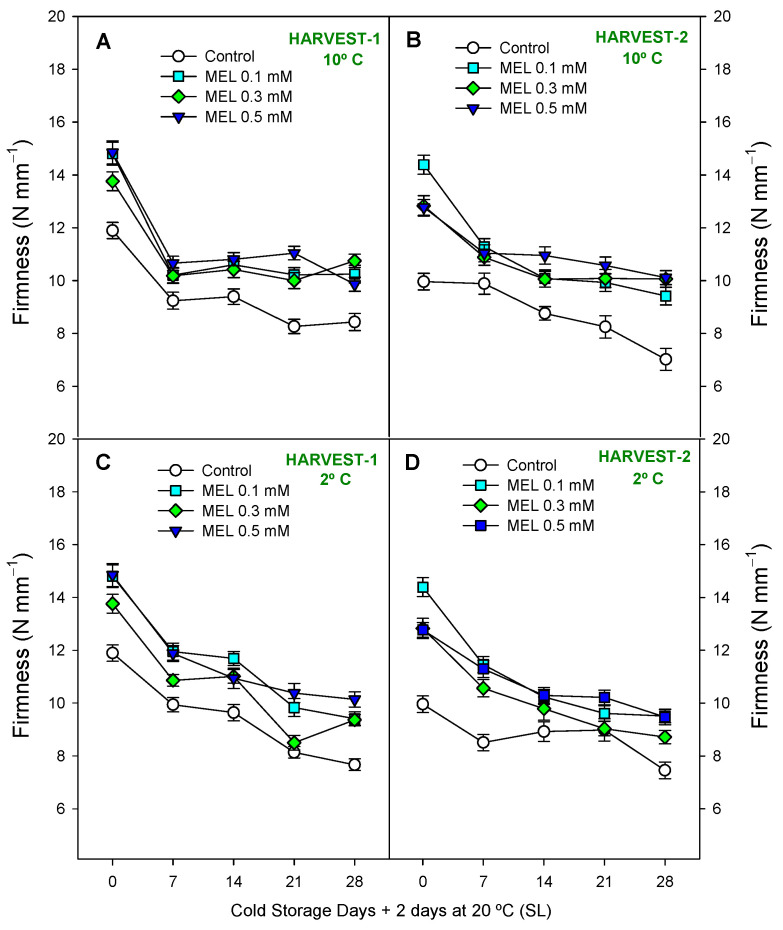
Firmness (N mm^−1^) during 28 days of storage + shelf-life (SL) at two temperatures (2 and 10 °C) from two harvest dates (HARVEST-1 and HARVEST-2) in lemon fruit as affected by melatonin (MEL) treatment. Data are the mean ± SE. LSD at *p* < 0.05 for the interaction treatment and storage time are not shown because these interactions were not significant.

**Figure 5:**
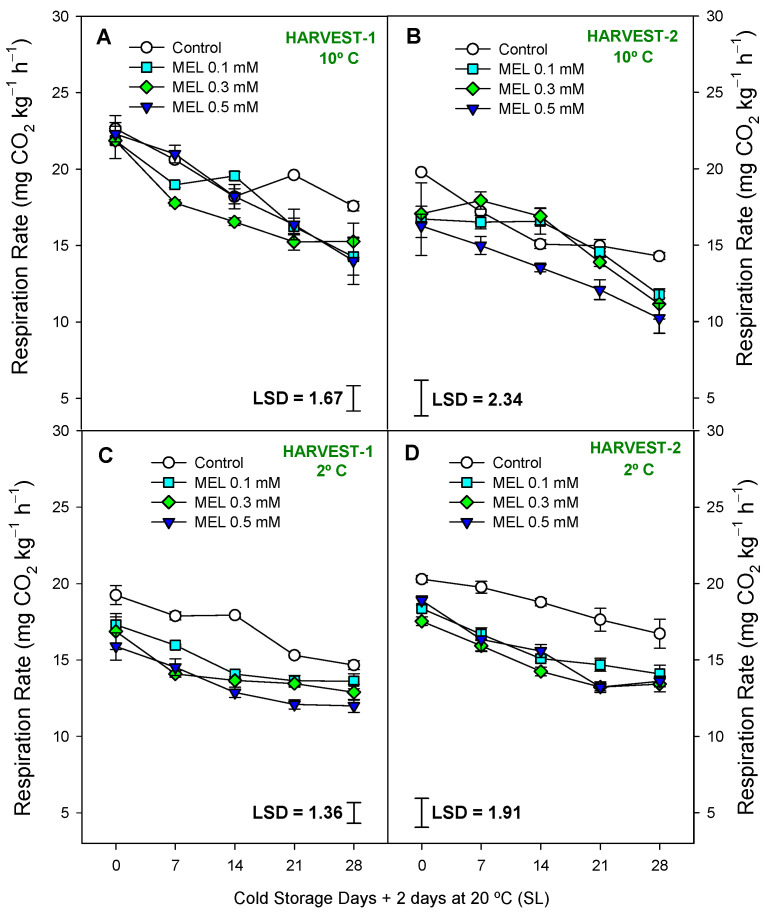
Respiration rate (mg CO_2_ kg^−1^ h^−1^) during 28 days of storage + shelf-life (SL) at two temperatures (2 and 10 °C) from two harvest dates (HARVEST-1 and HARVEST-2) in lemon fruit as affected by melatonin (MEL) treatment. Data are the mean ± SE. LSD at *p* < 0.05 for the interaction treatment and storage time are shown.

The next parameter studied was firmness. In Table S2, it can be observed that the MEL-treated lemons had higher firmness than the control fruits, being significantly higher at *p* < 0.05 in Figure 4A,C,D and significantly higher at *p* < 0.01 in Figure 4B. Overall, the most effective treatment for this parameter was MEL 0.5 mM. Firmness decreased significantly (*p* < 0.001) throughout storage, both at 2 and 10 °C, reaching losses of 30–40% compared to harvest time. No significant differences (*p* ≥ 0.05; Table S2) in the effect of the interaction between treatment and storage time were observed, so the LSD is not represented. Regarding the respiration rate, it can be observed in Table S2 and Figure 5A–D how all the factors, treatment and storage time and the interaction of both, showed significant differences with respect to the controls (*p* < 0.001), being the respiration rate of treated fruits lower than the control and with a decelerating evolution from the beginning to the end of cold storage in the four conditions of the study. In relation to total acidity (Figure S2) and total soluble solids (Figure S3), significant (*p* < 0.001, except harvest-2 at 10 °C with *p* < 0.05) effects of MEL treatments were observed (Table S2), with TA and TSS content showing higher values in fruit from MEL-treated trees with respect to control ones at the end of the cold storage in both harvests and temperatures. TSS showed a stable trend throughout storage, although the storage variable was significant (*p* < 0.001, except harvest-1 10 °C with *p* < 0.01) (Table S2). A similar behavior was observed with TA (*p* < 0.001; Table S2). Finally, it can be highlighted that in the TSS, the LSD, represented as the interaction between treatment and storage time, was significant (*p* < 0.001).

### 2.3. Fruit Functional Compounds and Antioxidant Activity

**Figure 6:**
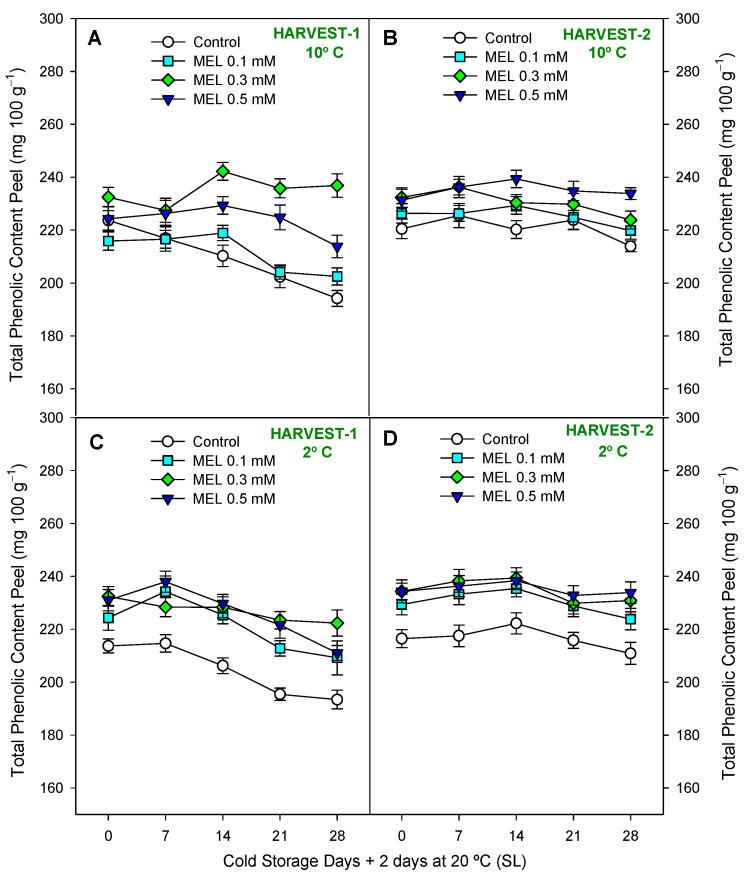
Total phenolic content in peel (mg·100 g^−1^) during 28 days of storage + shelf-life (SL) at two temperatures (2 and 10 °C) from two harvest dates (HARVEST-1 and HARVEST-2) in lemon fruit as affected by melatonin (MEL) treatment. Data are the mean ± SE. LSD at *p* < 0.05 for the interaction treatment and storage time point are not shown because such interaction were not significant.

**Figure 7:**
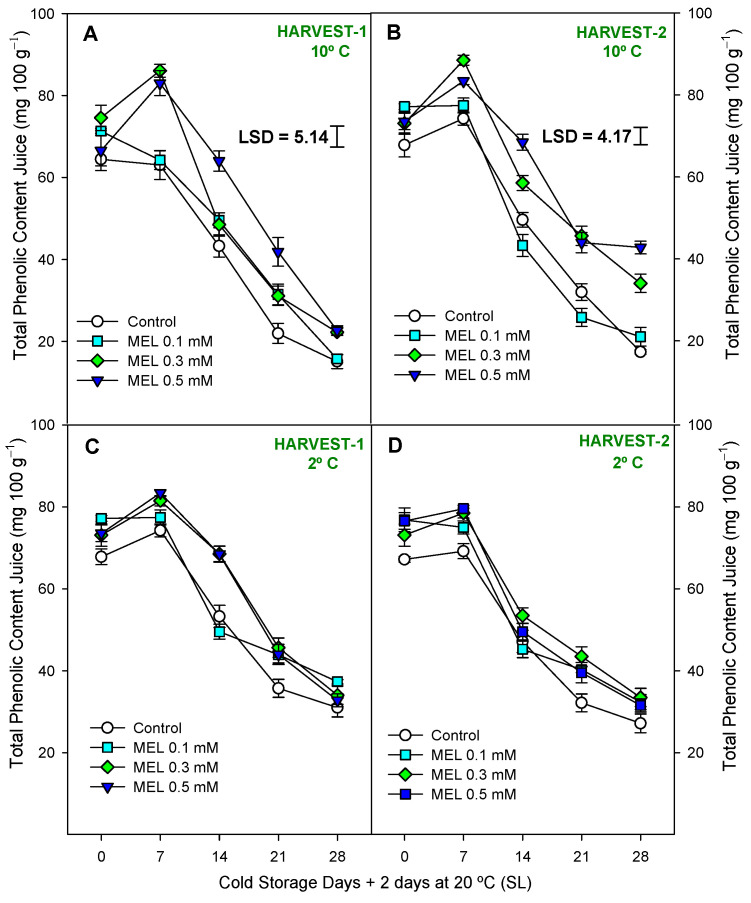
Total phenolic content in juice (mg 100 g^−1^) during 28 days of storage + shelf-life (SL) at two temperatures (2 and 10 °C) from two harvest dates (HARVEST-1 and HARVEST-2) in lemon fruit. Data are the mean ± SE. LSD at *p* < 0.05 for the interaction treatment and storage time are shown when such interaction was significant.

**Figure 8:**
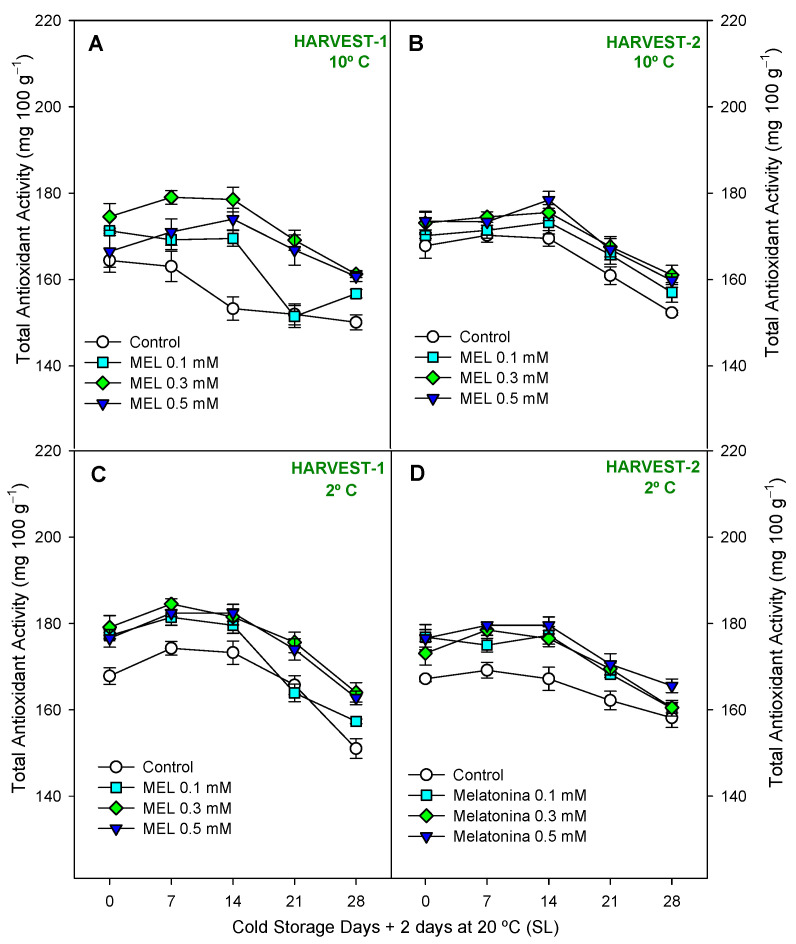
Total Antioxidant Activity (mg·100 g^−1^) during 28 days of storage + shelf-life (SL) at two temperatures (2 and 10 °C) from two harvest dates in lemon fruit. Data are the mean ± SE. LSD at *p* < 0.05 for the interaction treatment and storage time are shown when such interaction was significant.

In lemons, total phenolic content (TPC) was analyzed in both peel (Figure 6) and juice (Figure 7), and concentration was significantly higher in peel (≈200–250 mg 100 g^−1^) than in juice (20–40 mg 100 g^−1^) at the end of the preservation experiment at both harvests and temperatures. Also, they showed different behavior, with an increase during 28 days of storage in the peel and a decrease in the juice. However, either in TPC in peel or TPC in juice, it was shown a significant effect of melatonin treatment with respect to the control (Table S3). As for phenols of the peel, it can be observed that in both harvests and temperatures (Figure 6A–D), MEL at 0.3 and 0.5 mM led to significantly higher (*p* < 0.05) phenolic content than in control, in general, throughout the entire duration of the storage period. In turn, at 2 °C it was possible to observe (Table S3) higher significant differences (*p* < 0.01) between MEL-treatment and control. At 10 °C, in both harvest dates (Figure 7A,B), MEL-treated fruits had a significantly (*p* < 0.001; Table S3) higher TPC in juice than controls. At 2 °C, in both harvests (Figure 7C,D), significant differences were also observed (*p* < 0.05) with higher content in MEL-treated fruits. MEL 0.5 mM was the dose that led to the highest phenolic content in lemon juice at the end of storage at 10 °C, both in the first and second harvest, being 40% higher than the control at the same time. A drastic drop in the phenol content of the juice was observed during storage at both temperatures (Figure 7A–D), so we can say that storage time significantly influenced the loss of phenols from the juice (*p* < 0.001; Table S3). In Figure 8 and Table S3, we observe that the total antioxidant activity was significantly influenced (*p* < 0.05) by the treatment and storage time factors analyzed.

## 3. Discussion

Since the first pioneer report of preharvest MEL on pomegranate by García-Pastor et al. [34], few studies have addressed the role of MEL on crop yield, and results were contradictory depending on several factors, such as the number of applications, the stage of fruit development, and the fruit species, among others. To the best of our knowledge, this is the first evidence showing the efficacy of MEL on lemons during on-tree growth and ripening, resulting in enhancement of the yield performance of ‘Verna’ lemons, improving quality attributes at harvest, and maintenance after storage for 28 days at two temperatures (2 and 10 °C). Results of the present study clearly showed that MEL treatments of lemon trees produced increases in crop yield performance based on kg per tree and nº of fruits per tree. The highest effect was observed for the 0.5 mM dose, where the total yield improved by about 30% for the 1st and 2nd harvest dates compared to the control trees. In agreement with these results, MEL-treated sweet cherries, plums, or pomegranates with the same concentrations also increased crop yield [24,34,35] by enhancing the average fruit weight. Similarly, apricots treated with MEL at 0.1 mM showed a net increase in fruit yield at the time of harvest [25]. On the other hand, the application of MEL at other doses than those used in this study also showed positive effects on enhancing crop yield in strawberries and grape berries [36,37]. This yield potential could be related to two factors: the increase in the average fruit weight and the higher fruit number. In pomegranates, the increase in production was due to both factors [34]. The larger fruit size and weight could be the result of a higher accumulation of endogenous melatonin [37] or, alternatively, of a high photosynthetic efficiency and a delay in leaf senescence [38] and increases in net photosynthesis of the trees due to higher total chlorophyll and leaf area [25]. However, in the present study, the increase in yield was related to the higher number of fruits, since fruit weight did not show significant differences among treatments. Since treatments were applied at the exponential phase of the growth cycle, the observed higher fruit number in treated trees suggests a key role of MEL in reducing the fruitlet abscission or the “June-drop” occurring in citrus fruits, mainly due to environmental factors. In this sense, the role of MEL in counteracting the negative damage of both abiotic and biotic stressors has been reported [39,40,41]. It is well known that secondary metabolites such as phenolic compounds accumulate in fruit when plants are under abiotic stress conditions [42]. Then, taking into account that melatonin treatments increase total phenolic content in lemon fruit, fruit would be more tolerant to environmental stresses, such as wind, rain, or drought, leading to a reduction in the normal fruit drop during fruit development and to an increase in crop yield due to enhanced number of fruit harvest by tree. Therefore, MEL is thought to have influence on signal transduction as well as regulating plant physiological and biological processes, and thus considered as a biological plant growth regulator that improves a plant production capacity [43].

Appearance, together with internal quality, are important factors in determining the economic value of fruits and consumer preferences. Internal quality includes indicators of firmness, weight loss, respiration rate, TSS, and TA, among others. The results suggest that MEL application induced a retardation of the lemon ripening during the postharvest storage (at both temperatures), based on the delayed changes in respiration rate, weight loss, and fruit firmness by the action of preharvest MEL treatments. There is no scientific evidence regarding the role of preharvest MEL treatments on the behavior of these quality traits in lemon fruit. After 28 days of storage, control samples (untreated lemons) lost significantly more weight than the MEL-treated lemon fruits in both harvest and both temperatures. It is true that these losses were lower at 2 °C than at 10 °C. Postharvest weight loss in fruits and vegetables is caused by enhanced respiration and transpiration rates [44]. Weight loss increased as did the progression of storage [45], independently of the treatment, although, as expected, the 2 °C provoked lower physiological weight loss than 10 °C, which was attributed to the lower respiration rate and transpiration occurring at 2 °C [46,47]. These findings suggest that foliar application of MEL could improve preservation through a process involving reduction of the respiration rate [48], which agrees with the results of the present experiment, in which MEL treatments produced a reduction in respiration rate with respect to control fruits. Similar results were observed by Bal [49] on plum, who reported the least amount of weight loss in MEL treatments throughout storage as well as in ‘Balady Banzahir’ limes during shelf life [50]. 

Citrus fruits are susceptible to chilling injury (CI) when stored at low (non-optimal) temperature, although their susceptibility depends on fruit species and cultivar, apart from other agronomic or environmental factors during growth [51]. However, it is interesting to note that in the present experiment, no CI damage was found on the lemon peel surface until the last sampling date either in control, or in MEL-treated fruit. Accordingly, it has been reported that for the ‘Verna’ cultivar, the visual fruit appearance was good after four weeks of storage at 2 °C plus 2 days at 20 °C without external symptoms of CI damage [15]. However, in this previous paper, increases in ion leakage, which is an index used to measure membrane damage associated with CI, were reported, showing that membrane damage had started to occur, although it was not high enough to be manifested externally.

In terms of respiration rate, research in sweet cherries indicated that preharvest spraying with MEL diminished respiration rate at harvest time and during storage [52]. Regarding firmness, MEL treatments induced an important effect on delaying firmness losses at the end of storage for both temperatures and harvest dates. In several studies in which MEL was applied as a preharvest treatment, it was observed that ambiguous results depended on the dose and fruit species [24]. However, related to the postharvest of MEL-treated fruits, more results coinciding with the present ones were reported. For instance, the postharvest application of MEL in “Newhall” navel orange inhibited the firmness loss and kept it longer during storage [53], and similar effects were observed in citric “kiyomi tangor” [54]. 

For consumers, the contents of TSS and TA are considered essential quality traits related to acceptability. For this reason, MEL-treated lemons could be considered highly valued by consumers in comparison with control fruits. There are some reports evidencing that postharvest MEL could induce the suppression in the content of TSS or TA [31], although in tomato, MEL application stimulated the accumulation of TSS and particularly citric acid [55]. Consequently, it could be postulated that the reduction of the degradation rate of some nutritional compounds like TSS and TA, as well as the reduction of fruit softening, could be due to the delay of the normal postharvest maturation and senescence processes as a result of preharvest MEL treatments, leading to maintenance of lemon fruit quality attributes for longer periods. Similar findings were observed for guavas [52], oranges [53], and peaches [56] after postharvest MEL treatments. Moreover, postharvest MEL treatments of ‘Fino’ lemon fruit have been recently reported to have positive effects on reducing fruit weight loss, softening, color changes, and total acidity losses [57]. In this sense, Tijero et al. [58] found that the inhibition of postharvest ripening of melatonin-treated sweet cherry, could be related to the increased levels of cytokinins, suggesting a crosstalk between both plant hormones. However, this is not a single example since it has been reported in recent reviews that MEL could interact with other plant hormones, such as auxins, gibberellins, ABA, jasmonic acid and salicylic acid, as well as in other physiological processes involved in fruit ripening, such as degradation of cell wall components, carbohydrates and pigments, energy metabolism, and antioxidant systems, among others [41,59]. 

Citrus fruits are an excellent source of bioactive compounds considered as potent antioxidants, such as vitamin C and E, polyphenols, flavonoids, and carotenoids [3]. As can be seen in the obtained results, the flavedo of both the control and MEL-treated fruits has a higher concentration of phenolic compounds than the juice, showing that the peel of lemons and other citrus fruits, as an important source of phenolic compounds [60,61,62,63], could be very interesting as by-products to be revalorized in the food industry as a natural source of antioxidant compounds. The peel phenolic levels of control ‘Verna’ lemons found in this study were higher than those measured in peels of other lemon cultivars [64], showing genetic differences among lemon cultivars. On the other hand, our results agree with those obtained with limes where total phenolic content in the pulp decreased during storage [52], which could be attributed to the action of the enzyme polyphenol oxidase involved in the degradation of the phenolic compounds during the ripening process [61]. In similar studies on cherries and pomegranates, authors reported that total phenolic content increased after MEL treatment at 0.3 and 0.5 mM [24,37]. They found that MEL treatment enhanced glucose-6-phosphate dehydrogenase (G6H), shikimate dehydrogenase (SD), and phenylalanine ammonia lyase (PAL) activities but inhibited polyphenol oxidase (PPO) and peroxidase (POD) activities, which would lead to phenolics accumulation. On the other hand, an increase in phenolic content was also observed in MEL-treated oranges, suggesting that the capacity of resistance to oxidative stress was efficiently activated by MEL treatment through the enhancement of the ascorbate-glutathione cycle (AsA-GSH) [53]. Some studies showed a correlation between the increase in phenol content and the increase in the total antioxidant activity of the fruit [25,36] which is in line with the present results. Therefore, the higher antioxidant activity of the lemons treated with melatonin would be a consequence of the increase in phenolic compounds with antioxidant activity.

## 4. Materials and Methods

### 4.1. Plant Material, Experimental Design and Storage Conditions

The experiment was conducted in the growing season 2020–2021, in a commercial plot located in Orihuela (Alicante, Spain, 38°7′49.09″ N, 0°59′54.58″ W), under Mediterranean climate conditions (with ≈19 °C mean annual temperature and accumulated rainfall of 319 mm in 2021) and standard growing conditions for organic lemons. ‘Verna’ 15-year-old lemon trees grafted on *Citrus macrophylla* and planted at 7 × 5 m were randomly selected for each MEL concentration treatment. Melatonin (Sigma-Aldrich, Madrid, Spain) treatments were carried out by using 5 L per tree, applied with a foliar pulverization machine, of newly brewed melatonin solutions at 0.1, 0.3, and 0.5 mM containing 1 mL L^−1^ Tween 20. In the same way, 5 L of distilled water with 1 mL L^−1^ Tween 20 were applied to control trees. Each treatment utilized three replicates consisting of two trees. Every treatment was applied monthly, starting after the end of the physiological fruit drop stage until 3 days before the first harvest (1 February, 1 March, 31 March, and 30 April 2021). The fruits were harvested at the yellow commercial ripening stage, and since the on-tree maturation process of lemon fruit is not homogenous for all fruit, three harvests were performed: the first one on 3 May 2021(Harvest-1), the second on 31 May 2021 (Harvest-2), and the third one on 9 July 2021 (Harvest-3). In addition, treatments were repeated again 3 days before Harvest-2 and Harvest-3. For each harvest date, total yield per tree of commercial and non-commercial fruit was recorded as kg tree^−1^ and as number of fruits per tree (nº fruits tree^−1^). Non-commercial fruit or waste were separated according to commercial practices consisting of small fruit and fruit showing symptoms of fungal or pest attack and mechanical damages due to rubbing against branches or hail impact. In addition, a sample of 100 fruit per tree, for both commercial and waste fruit, was taken at random and weighed to obtain data on fruit weight average. For Harvest-1 and Harvets 2, these samples of 100 commercial fruit for each replicate were mixed and transported to the laboratory immediately. Then, 10 lots of 30 fruits, homogeneous in size and color, were selected for each of the three field replicates for each treatment. Four lots of each replicate were stored at 2 °C and 4 lots at 10 °C, with a relative humidity of 85–90%. At 7, 14, 21, and 28 days of storage, one lot of 30 fruit for each replicate and treatment was taken at random and left at 20 °C for two days to simulate commercial conditions after cold storage, in which the following analytical determinations were performed.

### 4.2. Physiological and Quality Parameters

After placing five fruits in a 0.5 L plastic bottle for 60 min, the respiration rate was measured at room temperature. Subsequently, 1 mL of the carrier atmosphere was taken and CO_2_ was quantified in a gas chromatograph (Shimadzu 14B-GC, Manchester, UK) coupled to a thermal conductivity detector [64], and the respiration rate was expressed as mg CO_2_ kg^−1^ h^−1^. Weight loss was expressed as a percentage (%) after calculating the difference between initial weight and weight after storage. The TX-XT2i texture analyzer (Stable Microsystems, Godalming, UK) coupled to a steel plate applied a force causing a deformation of 5% of the diameter of the fruit to measure the firmness, expressing the results in N mm^−1^. After these parameters were measured in each replicate, results were expressed as the mean ± SE. Lemon juice samples were then taken from each of the fruits comprising each replicate and combined, and the following parameters were measured: total soluble solids (TSS) using a digital refractometer (Hanna Instruments, RI, USA) and titratable acidity (TA) using an automatic titrator (785 DMP Titrino; Metrohm, Herisau, Switzerland), by titration of 0.5 mL of juice with 0.1 mM NaOH to pH 8.1; the results (mean ± SE) were expressed in % (g·100 mL^−1^).

### 4.3. Total Phenolics Content and Total Antioxidant Activity

Total phenols were measured in both peel (flavedo) and juice. For peel extraction, 2 g of flavedo were homogenized in 15 mL of water: methanol (2:8, *v*/*v*) containing 2.0 mM NaF. The extracts were centrifuged at 10,000× *g* for 15 min at 4 °C. For phenolic quantification in lemon juice, the extraction was similar. A total of 2 mL of juice was used and the centrifuge conditions were 8000× *g* for 7 min at 4 °C. Folin-Ciocalteau reagent was used to measure in duplicate the total phenolic content (TPC) in each sample, as previously reported [64]. Results (mean ± SE) were expressed as mg gallic acid equivalent 100 g^−1^ FW. The ABTS method was used to determine the total antioxidant activity. Briefly, 5 g of pulp were homogenized with 7.5 mL phosphate buffer (pH = 6.8) and 10 mL ethyl acetate for 2 min and centrifuged at 10,000× *g* at 4 °C for 20 min. After separation of the phases (hydrophilic and lipophilic), each extract was measured in duplicate with an ABTS peroxidase system and then summed and expressed as a total antioxidant activity in mg 100 g^−1^ Trolox equivalents. 

### 4.4. Statistical Analysis

For the field experiments, a randomized design of three replicates of two trees per treatment was used. Fruit samples were taken from each replicate and used for the storage experiment. The experimental data for each cultivar were independently subjected to ANOVA analysis. For yield analysis, the factors of variation were treatment and fruit type. For the storage experiment, the factors of variation were treatment and storage temperature. All analyses were performed with the SPSS v. 22.0 software package for Windows (SPSS, 2011). Finally, least significant differences (LSD) were calculated at *p* < 0.05 for the interaction between the analyzed factors, and the values are shown in each figure.

## 5. Conclusions

The results of this study showed that MEL, applied as preharvest treatment in ‘Verna’ lemon trees along the growth cycle, was effective on improving the crop performance and yield since a higher percentage of commercial lemons and fewer wasted fruits were obtained, the best concentration being 0.5 mM. In addition, quality traits at harvest (fruit firmness and the content of TSS and TA) were higher in MEL-treated ‘Verna’ lemons in the two harvests (harvest-1 and harvest-2) in which the yield and quality of fruit were analyzed, lasting during storage. Moreover, MEL-treated trees showed a higher concentration of total phenolic compounds in juice as well as in the peel of lemons, especially for the 0.5 mM of MEL. Considering all quality traits, we can conclude that MEL induced a certain delay in the postharvest senescence at 2 °C (two weeks) and 10 °C (one week). For this reason, we demonstrated preharvest treatments with MEL may be effective in increasing crop yield to obtain higher-quality lemons. This can be regarded as a natural and environmentally friendly strategy and could alleviate the negative effects of climate change and, more generally, of abiotic stresses. Nevertheless, the physiological mechanisms involved in the effects of MEL preharvest treatments on increasing lemon tree yield and retarding fruit quality losses during storage deserve future research.

## Data Availability

The original contributions generated for this study are included in the article; the data presented in this study are available on request from the authors.

## Supplementary Materials

The following supporting information can be downloaded at: https://www.mdpi.com/article/10.3390/ijms251810025/s1.
