# Supplementary material for: Melatonin as an Efficient and Eco-Friendly Tool to Increase Yield and to Maintain Quality Attributes during Lemon Storage
Source: Int J Mol Sci. 2024 Sep 18;25(18):10025. doi: 10.3390/ijms251810025 (PMC11432733; doi:10.3390/ijms251810025)
Supplement: Supplementary file 1 [file ijms-25-10025-s001.zip › ijms-3219700-supplementary.pdf]

International Journal of *Molecular Sciences*

MDPI

Article

Melatonin as an Efficient and Eco-Friendly Tool to Increase Yield and to Maintain Quality Attributes during Lemon Storage

Fátima Badiche-El Hilali [1], María E. García-Pastor [2], Juan Miguel Valverde [1], Salvador Castillo [1], Daniel Valero [1,*] and María Serrano [2,*]

[1] Department of AgroFood Technology, Escuela Politécnica Superior de Orihuela (EPSO), Instituto de Investigación e Innovación Agroalimentario y Agroambiental (CIAGRO), University Miguel Hernández, Ctra. Beniel km. 3.2, Orihuela, 03312 Alicante, Spain; fbadiche@umh.es (F.B.-E.H.); jm.valverde@umh.es (J.M.V.); scastillo@umh.es (S.C.)

[2] Department of Applied Biology, Escuela Politécnica Superior de Orihuela (EPSO), Instituto de Investigación e Innovación Agroalimentario y Agroambiental (CIAGRO), University Miguel Hernández, Ctra. Beniel km. 3.2, Orihuela, 03312 Alicante, Spain; m.garciap@umh.es

* Correspondence: Correspondence: daniel.valero@umh.es (D.V.); m.serrano@umh.es (M.S.)

Table S1. Analyses of variance (ANOVA) of crop parameters (kg tree^{-1}, fruit number tree^{-1} and fruit weight) for different harvest dates (1st, 2nd and 3rd harvest) as according to 2 categories: commercial and waste in 'Verna' lemon using the treatment, the production type and the interaction between the two as factors.

Parameter	Harvest-Nº	Treatment	Production type	Treatment * Production type	Reference
Yield (kg tree^{-1})	Harvest-1	32.98***	948.06***	97.06***	Fig. 1A
Yield (kg tree^{-1})	Harvest-2	3.39*	1075.85***	5.56**	Fig. 1B
Yield (kg tree^{-1})	Harvest-3	0.26 (NS)	216.41***	0.97 (NS)	Fig. 1C
Fruit number tree^{-1}	Harvest-1	3.44*	854.58***	4.00*	Fig. 2A
Fruit number tree^{-1}	Harvest-2	3.87*	1141.27***	4.07*	Fig. 2B
Fruit number tree^{-1}	Harvest-3	18.62***	457.00***	19.32***	Fig. 2C
Fruit weight (g)	Harvest-1	0.50 (NS)	1.71 (NS)	1.03 (NS)	Fig. S1A
Fruit weight (g)	Harvest-2	0.99 (NS)	2.66 (NS)	1.52 (NS)	Fig. S1B
Fruit weight (g)	Harvest-3	0.70 (NS)	1.80 (NS)	1.33 (NS)	Fig. S1C

[1] NS = not significant; *, ** and *** significant at $p < 0.05$, $p < 0.01$ and $p < 0.001$, respectively; data were previously tested for normality test. Production type is classified as: commercial or waste

Table S2. Analyses of variance (ANOVA) of quality parameters [weight loss, respiration rate (RR), firmness, total soluble solids (TSS) and total acidity (TA)] for different harvest dates (1st and 2nd) in 'Verna' lemon during 28 days of storage + shelf-life at 2 temperatures (2 and 10 °C) using the treatment, the storage time and the interaction between the two as factors.

Parameter	Harvest-Nº	Temperature	Treatment	Storage time	Treatment * Storage time	Reference
Weight Loss (%)	Harvest-1	10 °C	49.15***	1915.78***	5.74***	Fig. 3A
Weight Loss (%)	Harvest-2	10 °C	15.80***	701.75***	1.99*	Fig. 3B
Weight Loss (%)	Harvest-1	2 °C	13.72***	359.69***	2.92**	Fig. 3C
Weight Loss (%)	Harvest-2	2 °C	28.12***	709.56***	3.59***	Fig. 3D
Firmness (N mm^{-1})	Harvest-1	10 °C	3.58*	10.93***	0.58 (NS)	Fig. 4A
Firmness (N mm^{-1})	Harvest-2	10 °C	4.17**	7.43***	0.69 (NS)	Fig. 4B
Firmness (N mm^{-1})	Harvest-1	2 °C	3.73*	8.07***	0.56 (NS)	Fig. 4C
Firmness (N mm^{-1})	Harvest-2	2 °C	3.82*	7.60***	0.59 (NS)	Fig. 4D
RR (mg CO_2 kg^{-1} h^{-1})	Harvest-1	10 °C	25.37***	125.16***	14.22***	Fig. 5A
RR (mg CO_2 kg^{-1} h^{-1})	Harvest-2	10 °C	7.06***	7.33***	8.32***	Fig. 5B
RR (mg CO_2 kg^{-1} h^{-1})	Harvest-1	2 °C	5.97***	93.66***	10.75***	Fig. 5C
RR (mg CO_2 kg^{-1} h^{-1})	Harvest-2	2 °C	2.08 (NS)	35.33***	11.87***	Fig. 5D
TSS (g 100 g^{-1})	Harvest-1	10 °C	32.58***	4.88**	8.71***	Fig. S2A
TSS (g 100 g^{-1})	Harvest-2	10 °C	9.66***	9.76***	9.34***	Fig. S2B
TSS (g 100 g^{-1})	Harvest-1	2 °C	59.43***	7.39***	4.98***	Fig. S2C
TSS (g 100 g^{-1})	Harvest-2	2 °C	52.58***	53.24***	13.54***	Fig. S2D
TA (%)	Harvest-1	10 °C	7.55***	11.99***	0.915 (NS)	Fig. S3A
TA (%)	Harvest-2	10 °C	3.88*	10.47***	0.90 (NS)	Fig. S3B
TA (%)	Harvest-1	2 °C	6.81***	25.52***	1.29 (NS)	Fig. S3C
TA (%)	Harvest-2	2 °C	13.95***	22.31***	1.51 (NS)	Fig. S3D

[1] NS = not significant; *, ** and *** significant at $p < 0.05$, $p < 0.01$ and $p < 0.001$, respectively; data were previously tested for normality test.

Table S3. Analyses of variance (ANOVA) of functional parameters [total phenolic content in peel (TPC peel), total phenolic content in juice (TPC juice) and total antioxidant activity (TAA)] for different harvest dates (1st and 2nd) in 'Verna' lemon during 28 days of storage + shelf-life at 2 temperatures (2 and 10 °C) using the treatment, the storage time and the interaction between the two as factors.

Parameter	Harvest-Nº	Temperature	Treatment	Storage time	Treatment * Storage time	Reference
TPC Peel (mg 100 g^{-1})	Harvest-1	10 °C	3.43*	18.68***	0.24 (NS)	Fig. S4A
TPC Peel (mg 100 g^{-1})	Harvest-2	10 °C	3.08*	17.51***	0.25 (NS)	Fig. S4B
TPC Peel (mg 100 g^{-1})	Harvest-1	2 °C	10.14**	20.08***	0.31 (NS)	Fig. S4C
TPC Peel (mg 100 g^{-1})	Harvest-2	2 °C	10.04**	17.13***	0.27 (NS)	Fig. S4D
TPC Juice (mg 100 g^{-1})	Harvest-1	10 °C	23.92***	486.32***	5.64***	Fig. 6A
TPC Juice (mg 100 g^{-1})	Harvest-2	10 °C	13.14***	243.32***	1.87*	Fig. 6B
TPC Juice (mg 100 g^{-1})	Harvest-1	2 °C	3.18*	186.19***	1.76 (NS)	Fig. 6C
TPC Juice (mg 100 g^{-1})	Harvest-2	2 °C	2.73*	213.66***	0.90 (NS)	Fig. 6D

TAA (mg 100 g^{-1})	Harvest-1	10 °C	3.18*	20.12***	0.36 (NS)	Fig. S5A
TAA (mg 100 g^{-1})	Harvest-2	10 °C	3.25*	21.70***	0.25 (NS)	Fig. S5B
TAA (mg 100 g^{-1})	Harvest-1	2 °C	3.73*	23.79***	0.26 (NS)	Fig. S5C
TAA (mg 100 g^{-1})	Harvest-2	2 °C	3.12*	20.88***	0.32 (NS)	Fig. S5D

[1] NS = not significant; *, ** and *** significant at $p < 0.05$, $p < 0.01$ and $p < 0.001$, respectively; data were previously tested for normality test.

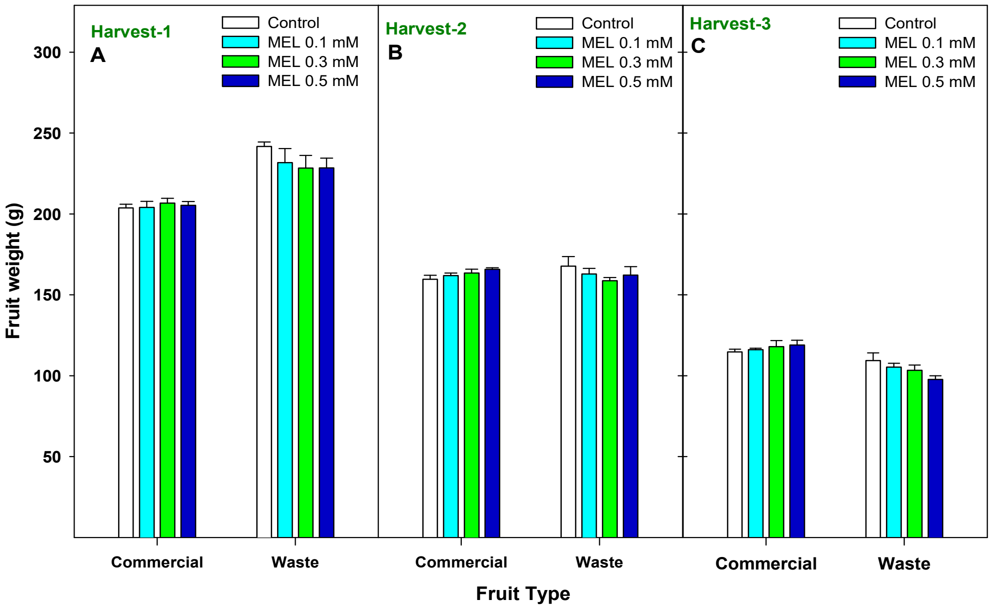

Figure S1. Influence of Melatonin preharvest treatment on yield (average fruit weight) in 3 harvest dates as according to 2 categories: commercial and waste. Data are the mean ± SE. LSD at $p < 0.05$ for the interaction treatment*production type point is only shown when such interaction was significant.

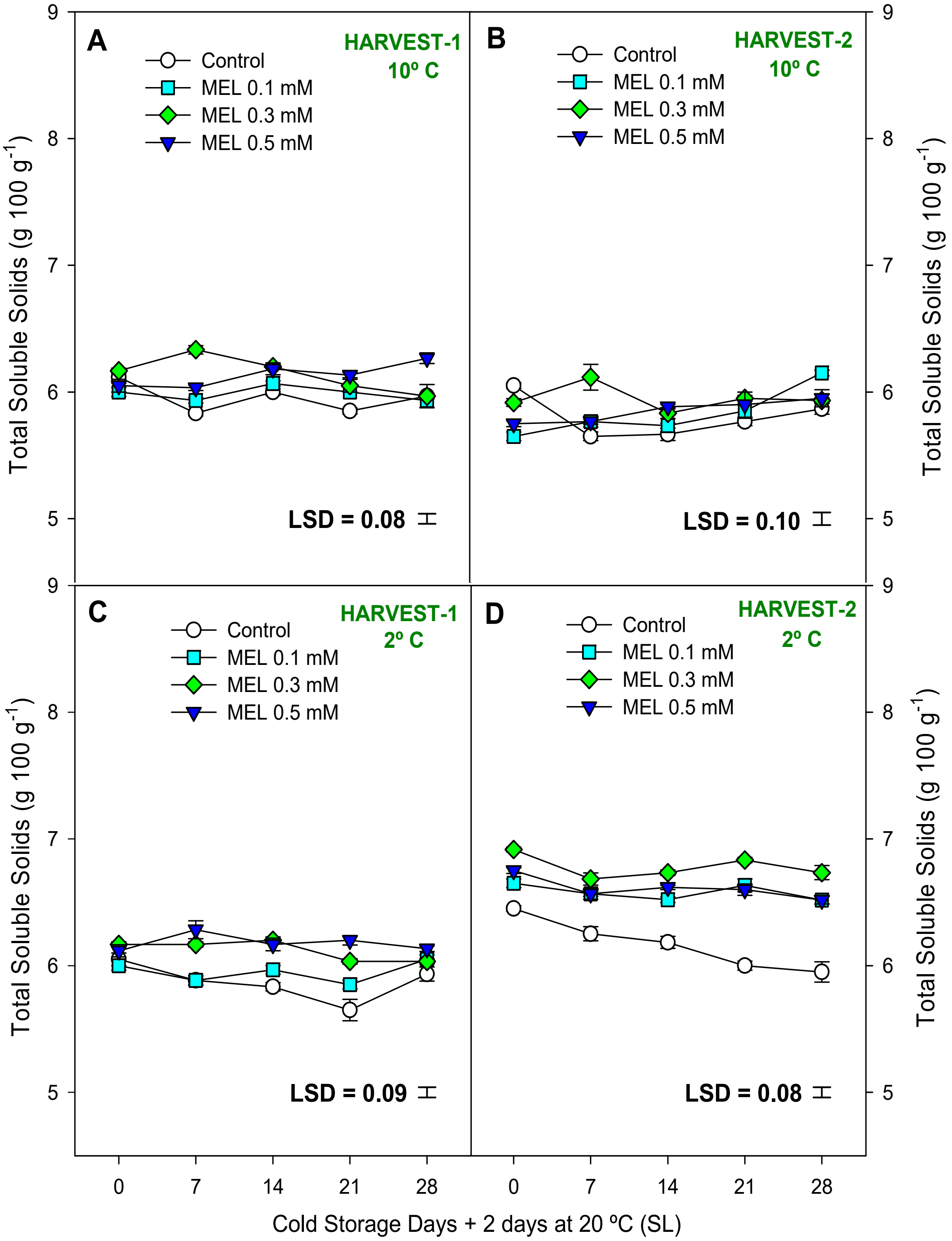

Figure S2. Total Soluble Solids (g 100 g^{-1}) during 28 days of storage + shelf-life (SL) at 2 temperatures (2 and 10 °C) from 2 harvest dates in lemon fruit. Data are the mean ± SE. LSD at $p < 0.05$ for the interaction treatment*storage time point is only shown when such interaction was significant.

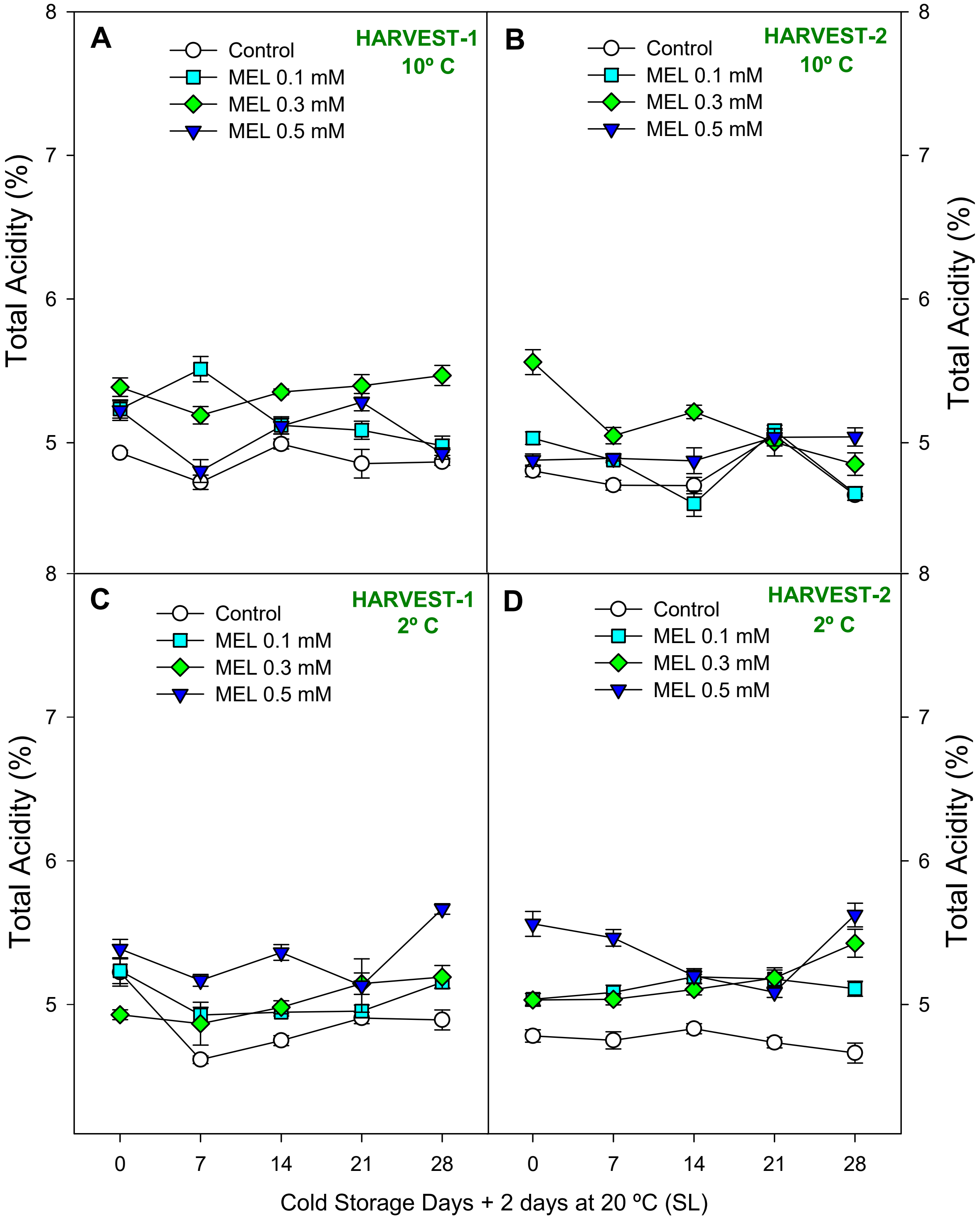

Figure S3. Total Acidity (%) during 28 days of storage + shelf-life (SL) at 2 temperatures (2 and 10 °C) from 2 harvest dates in lemon fruit. Data are the mean ± SE. LSD at $p < 0.05$ for the interaction treatment*storage time point is only shown when such interaction was significant.
